# CD44 regulates prostate cancer proliferation, invasion and migration via PDK1 and PFKFB4

**DOI:** 10.18632/oncotarget.17821

**Published:** 2017-05-11

**Authors:** Wei Li, Li Qian, Junhao Lin, Guihai Huang, Nan Hao, Xiuwang Wei, Wei Wang, Jianbo Liang

**Affiliations:** ^1^ Department of Urology, The People’s Hospital of Guangxi Zhuang Autonomous Region, NanNing, China; ^2^ Department of Pharmacy, The People’s Hospital of Guangxi Zhuang Autonomous Region, NanNing, China

**Keywords:** prostate cancer, CD44, PDK1, PFKFB4, metabolism

## Abstract

Our recent studies have shown that CD44, a cell-surface protein with functions in many biologic processes, involved in glucose metabolism of prostate cancer cells. However, the molecular mechanisms of the regulation need to be further elucidated. In present study, LNCaP cells infected with lentivirus vector overexpressing CD44. The expression levels of key enzymes in glucose metabolism known as PDK1 and PFKFB4 were determined using QRT-PCR and western blot. PDK1 and PFKFB4 in LNCaP and PC3 cells were knocked down with shRNA respectively, and then cell proliferation, invasion and cell migration assay were performed. We found that overexpression of CD44 increased expression levels of PDK1 and PFKFB4 in LNCaP cells. Silencing of PDK1 and PFKFB4 could decrease cell proliferation, inhibit invasion and migration ability of prostate cancer cells. In addition, CD44 inhibitor could decrease glucose consumption and increase ROS levels of PC-3 cells significantly, as well as sensitize PC-3 cells to docetaxel. Taken together, CD44 could modulate aggressive phenotype of prostate cancer cells, by regulation of the expression of PDK1 and PFKFB4. CD44 may be a novel potential therapeutic target.

## INTRODUCTION

Prostate cancer (PCa) is the most common cancer in men in western countries. It is estimated that there will be 180,890 new cases and lead to 26,120 deaths in the United States in 2016 [1]. However, the incidence and mortality of prostate cancer have been increasing significantly in China in past decades. The data from The National Central Cancer Registry of China indicated the estimated incidence and mortality for prostate cancer were 60.3 and 26.6 per 100,000 population in 2015 [2].

In contrast to benign cells, cancer cells depend primarily on the relatively inefficient glycolysis for their energy production, and the increased glycolysis of cancer cells is known as Warburg effect [3]. Although this phenomenon was described nearly hundred years ago, the exact mechanism of Warburg effect remains unclear. The metabolism of cancer attracted renewing attention recently due to widespread clinical application of Fluorodeoxyglucose (FDG) -Positron Emission Tomography (PET) imaging which is based on elevated glucose uptake by cancer cells.

CD44 is a multifunctional cell membrane receptor involved in cell adhesion, tumor invasion and metastasis. It also has emerged as a cancer initiating or stem cells [4]. Previous studies had demonstrated that CD44 could modulate glycolytic pathway in colorectal cancer and lung carcinoma through interacting with pyruvate kinase M2 [5]. Our most recently published study indicated CD44 regulated glucose metabolism, intracellular reactive oxygen species(ROS), and cell proliferation in PC3 cells.[6] 6-phosphofructo-2-kinase/fructose-2,6-biphosphatase 4 (PFKFB4) is required for balancing glycolytic activity and antioxidant production in prostate cancer cells [7]. Compare with naïve AdenoCa LNCaP and VCaP cells, PC3 and DU145 cells express relatively higher levels of PFKFB4 [6]. Similarly, CD44 was expressed in PC3 cells but not in LNCaP cells [8]. Several studies have shown that expression levels of metabolic enzymes involved in aerobic glycolysis of cancer cells are usually associated with aggression of tumor [9] [10]. The facts above raise a possibility that CD44 plays a critical role in glucose metabolism via regulation of downstream metabolic genes, which probably is involved in tumor proliferation, invasion and migration.

## RESULTS

### Expression of PDK1 and PFKFB4 in prostate cancer cells regulated by CD44

**Figure 1:**
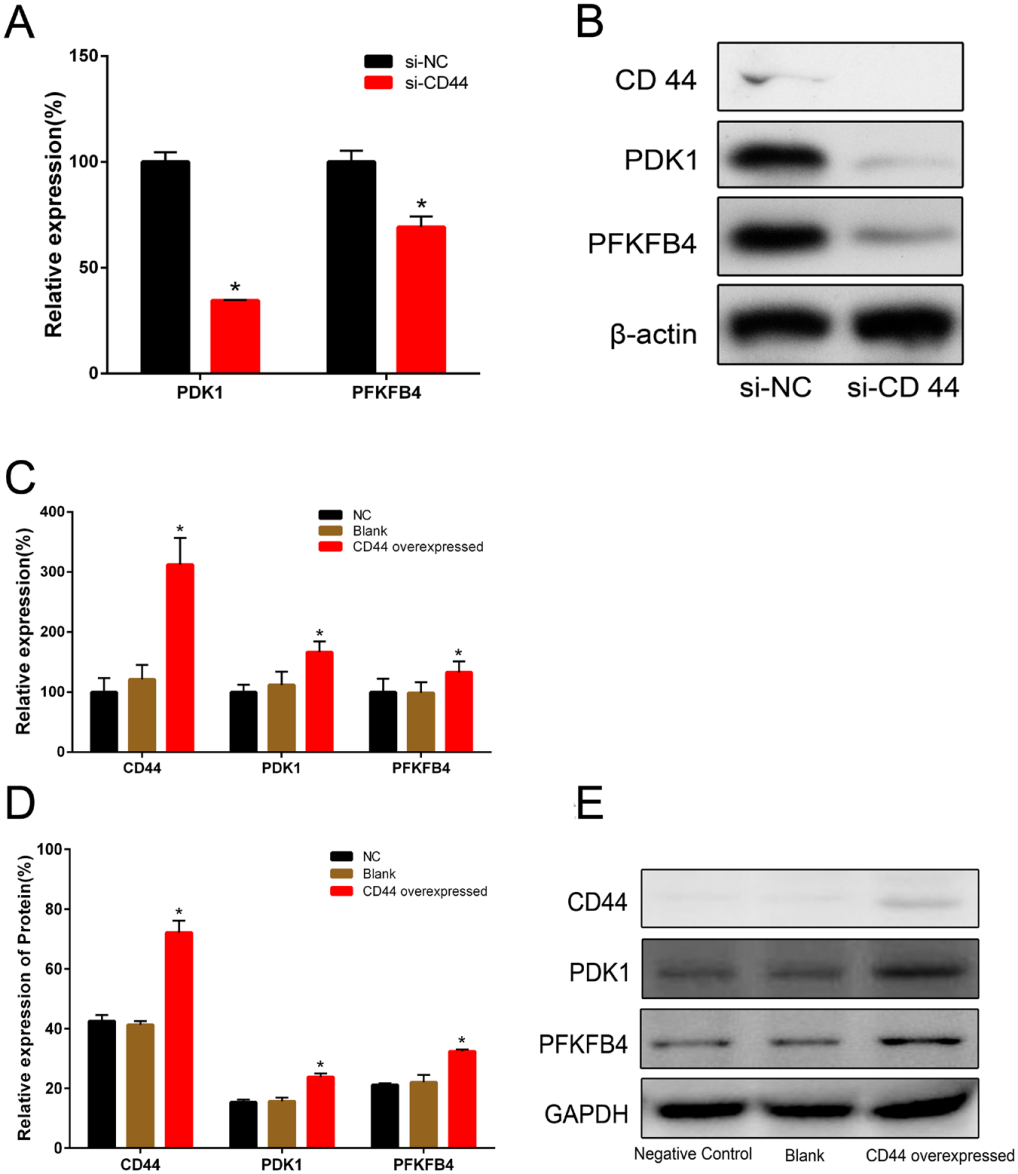
The relative expressions of PDK1 and PFKFB4 in mRNA and protein after CD44 knockdown in PC-3 cells and CD44 overexpression in LNCaP cells The relative expressions of PDK1 and PFKFB4 mRNA were decreased in si-CD44 group compared to si-NC group in PC-3 cells **(A)**. Data are shown as mean±SD (* p<0.05). Western blots detection for CD44, PDK1 and PFKFB4 expression in si-CD44 group and si-NC group of PC-3 cells **(B)**. β-actin was used as a reference. The relative expressions of CD44, PDK1 and PFKFB4 mRNA were increased in the group of CD44 overexpression compared to the group of NC **(C)**. Overexpression of CD44 lead to increased expression of CD44, PDK1 and PFKFB4 proteins in LNCaP cells **(D)**. Data are shown as mean±SD (* p<0.05). Western blots detection for CD44, PDK1 and PFKFB4 expressions in NC, Blank and CD44 overexpression group of LNCaP cells **(E)**. GAPDH was used as a reference.

We had demonstrated CD44 played a critical role in alteration of glucose metabolism [6]. As a glycolytic regulator, we examined whether CD44 affected the expressions of glycolytic enzymes involved in Kreb’s cycle. After CD44 knockdown by siRNA, we found significantly decreased expressions of PDK1 and PFKFB4 in both mRNA (Figure 1A) and protein (Figure 1B) in PC-3 cells. Similarly, when we overexpressed CD44 in LNCaP cells, compared with blank and negative control, we found the mRNA levels of PDK1 and PFKFB4 were increased significantly (Figure 1C), as well as relative expression of protein (Figure 1D, 1E).

### PDK1 and PFKFB4 regulate the proliferation, migration and invasion of prostate cancer cells *in vitro*

According to FDG-PET imaging of tumor patients, cancer cells showed significantly increased glucose uptake. Cells derived from tumors usually keep their metabolic phenotypes, and the glycolytic rate of cultured cell lines seems to link with tumor aggressiveness. For example, non-invasive MCF-7 breast cancer cells have much lower aerobic glucose consumption rate compared to the highly invasive MDA-mb-231 breast cancer cell line [11]. It was well known the enzymes in the metabolic pathways, including glycolysis, glutaminolysis and fatty acid biosynthesis, have been established as important for the survival and growth of cancer cells, then we evaluated the effects of PDK1 and PFKFB4 on aggressive phenotypes including proliferation, migration and invasion.

**Figure 2:**
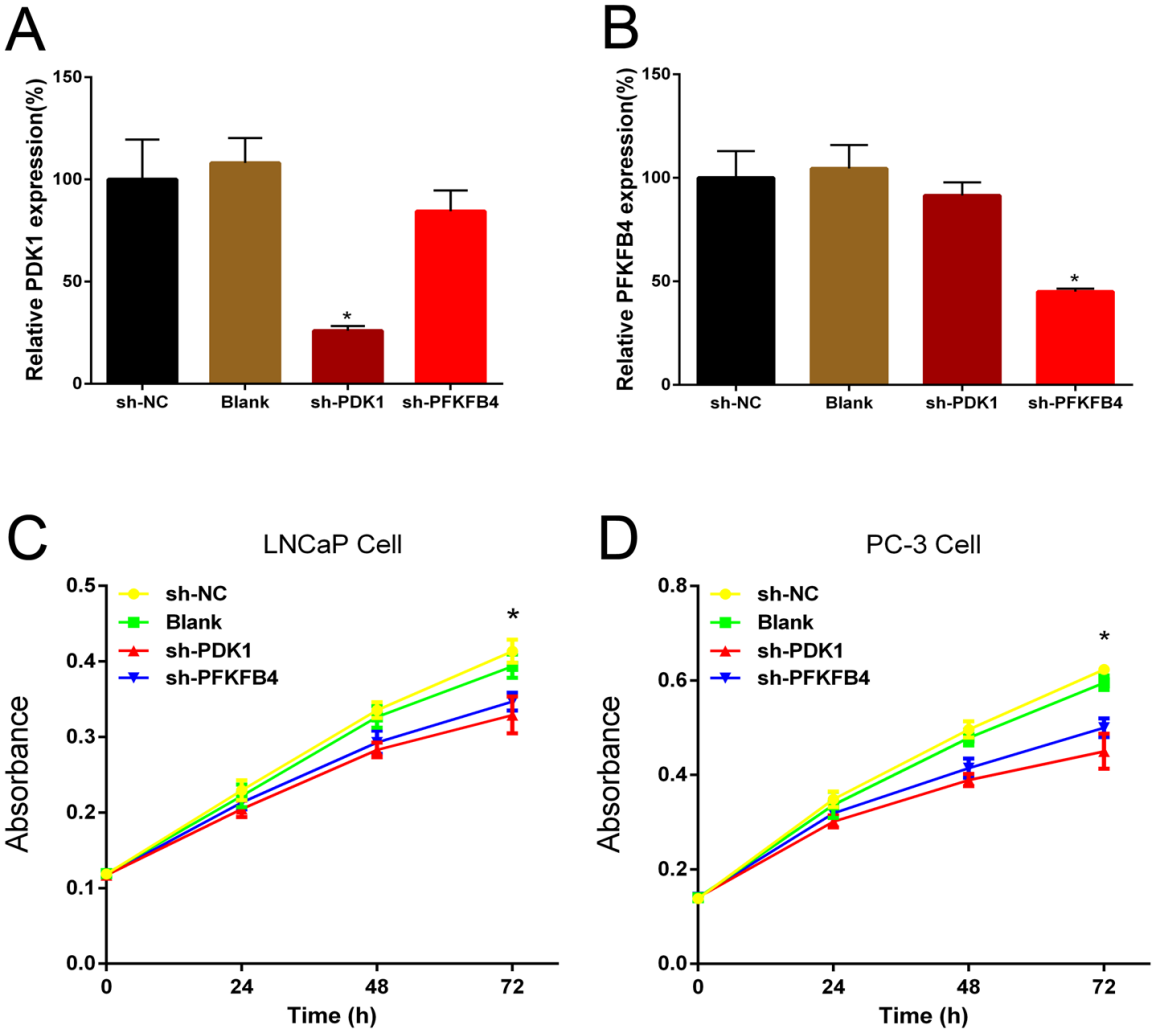
Short hairpin RNA PDK1 (sh-PDK1) and PFKFB4 (sh-PFKFB4) suppressed PDK1 and PFKFB4 mRNA expression respectively in LNCaP cells. Down-regulation of PDK1 and PFKFB4 inhibited cell proliferation in LNCaP cells and PC-3 cells QRT-PCR shown that PDK1 and PFKFB4 were efficiently knocked down by shRNA. The relative expression of PDK1 and PFKFB4 mRNA were decreased significantly in sh-PDK1 group **(A)** and sh-PFKFB4 group **(B)** respectively compared to blank and NC group in LNCaP cells. Data are shown as mean±SD (* p<0.05). Cell proliferation was suppressed in sh-PDK1 group and sh-PFKFB4 compared to sh-NC group in LNCaP cells **(C)** and PC-3 cells **(D)** by using MTT assay. Data are shown as mean±SD (* p<0.05).

First, we investigated the effects of PDK1 and PFKFB4 for prostate cancer cell proliferation using MTT assay. When we knocked down the PDK1 and PFKFB4 in LNCaP and PC3 cells, and the effects of knockdown were confirmed with QRT-PCR assay (Figure 2A and 2B). The results indicated depletion of PFKFB4 decreased cell proliferation significantly compared with the blank group and negative control group. Similarly, PDK1 silencing significantly inhibited the cell proliferation in both LNCaP (Figure 2C) and PC3 cells (Figure 2D).

**Figure 3:**
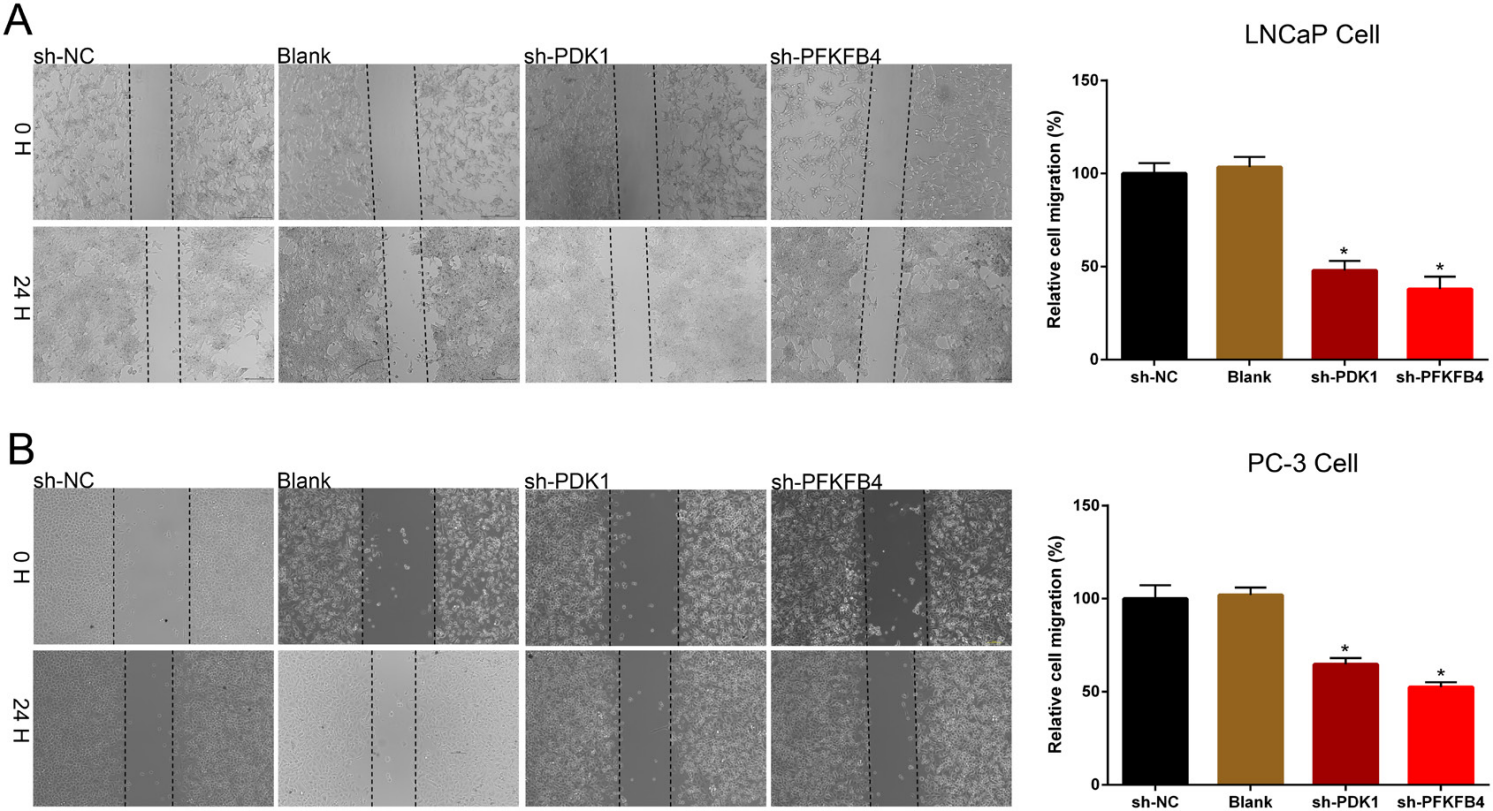
Down-regulation of PDK1 and PFKFB4 inhibited cell migration in LNCaP cells and PC-3 cells The rate of migration was decreased in sh-PDK1 group and sh-PFKFB4 group compared to sh-NC group in LNCaP cells **(A)** and PC-3 cells **(B)** by using wound-healing assay. Data are shown as mean±SD (* p<0.05).

Next, wound healing assays were performed to measure the migration of cells. We found the prostate cancer cells which transfected with PDK1 or PFKFB4 shRNA obtained slower closure of the scratched wound, and the relative cell migration of LNCaP (Figure 3A) and PC3 cells (Figure 3B) were decreased significantly compared with the cells infected negative vector control cells and blank cells. These results suggested depletion of PDK1 or PFKFB4 inhibited cell migration and motility.

**Figure 4:**
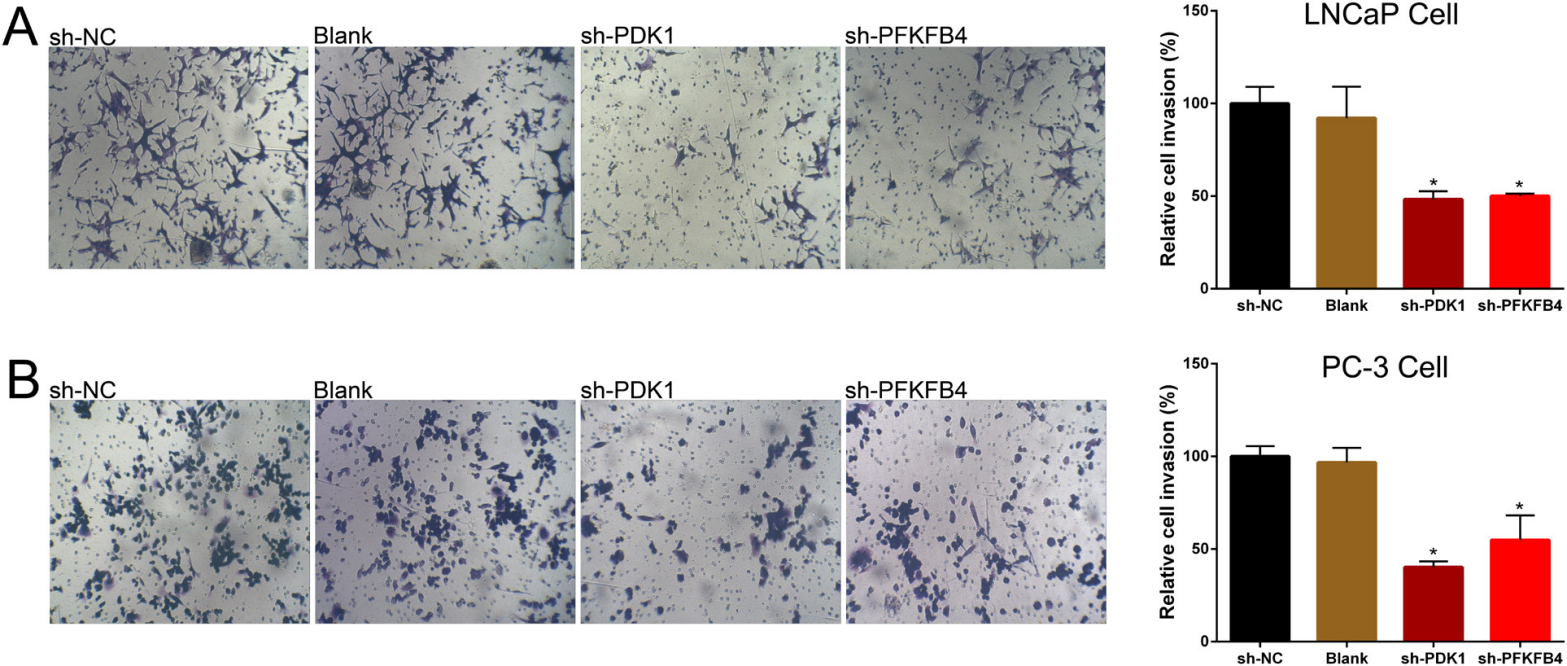
Down-regulation of PDK1 and PFKFB4 inhibited cell invasion in LNCaP cells and PC-3 cells Invasion of cells was suppressed in sh-PDK1 group and sh-PFKFB4 group compared to sh-NC group in LNCaP cells **(A)** and PC-3 cells **(B)** by using transwell assay. Data are shown as mean±SD (* p<0.05).

Finally, cell invasion was tested by using Transwell assay. The results indicated the invasion ability of PDK1 or PFKFB4 knockdown cells of LNCaP (Figure 4A) and PC3 cells (Figure 4B) were significant suppressed compared to blank cells and negative control cells.

### The effects of matrix metalloproteinase inhibitor (**SB-3CT**) on glucose consumption, ROS and genes expression

**Figure 5:**
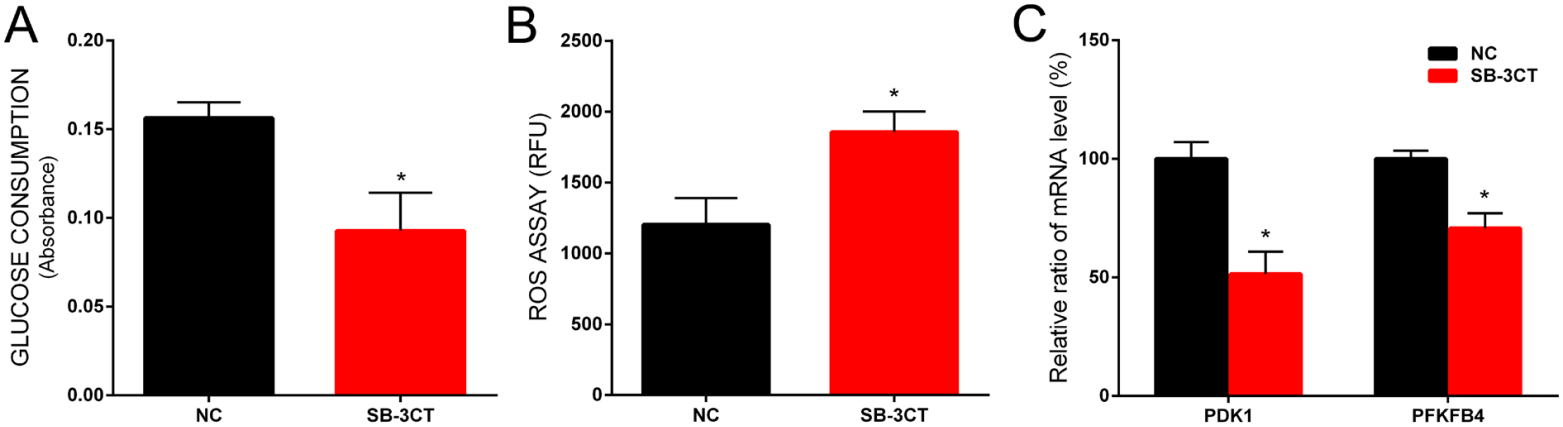
The effect of SB-3CT on glucose consumption, ROS level and gene expression in PC-3 cells The glucose consumption was decreased in PC-3 cells treated with SB-3CT **(A)**. The ROS level of PC-3 cells was increased in PC-3 cells treated with SB-3CT **(B)**. The relative expression of PDK1 and PFKFB4 mRNA was decreased in PC-3 cells treated with SB-3CT **(C)**. Data are shown as mean±SD (* p<0.05).

To further confirm the central role of CD44 in the glycolytic activity of prostate cancer cells, we evaluated glucose consumption and intracellular ROS levels in PC-3 cells treated with SB-3CT, a highly selective inhibitor known to target only matrix metalloproteinase 2 (MMP- 2) and matrix metalloproteinase 9 (MMP-9)[12]. It has been reported that MMP inhibitors could block CD44 cleavage and inhibit downstream signaling pathway [13]. We showed that treatment with SB-3CT decreased glucose consumption (Figure 5A) and increased ROS levels of PC-3 cells significantly (Figure 5B). In addition, SB-3CT treatment, similar to CD44 knockdown, reduced the expression of PDK1 and PFKFB4 significantly (Figure 5C).

### The synergistic effect of matrix metalloproteinase inhibitor combined with docetaxel on PC-3 cells viability

**Figure 6:**
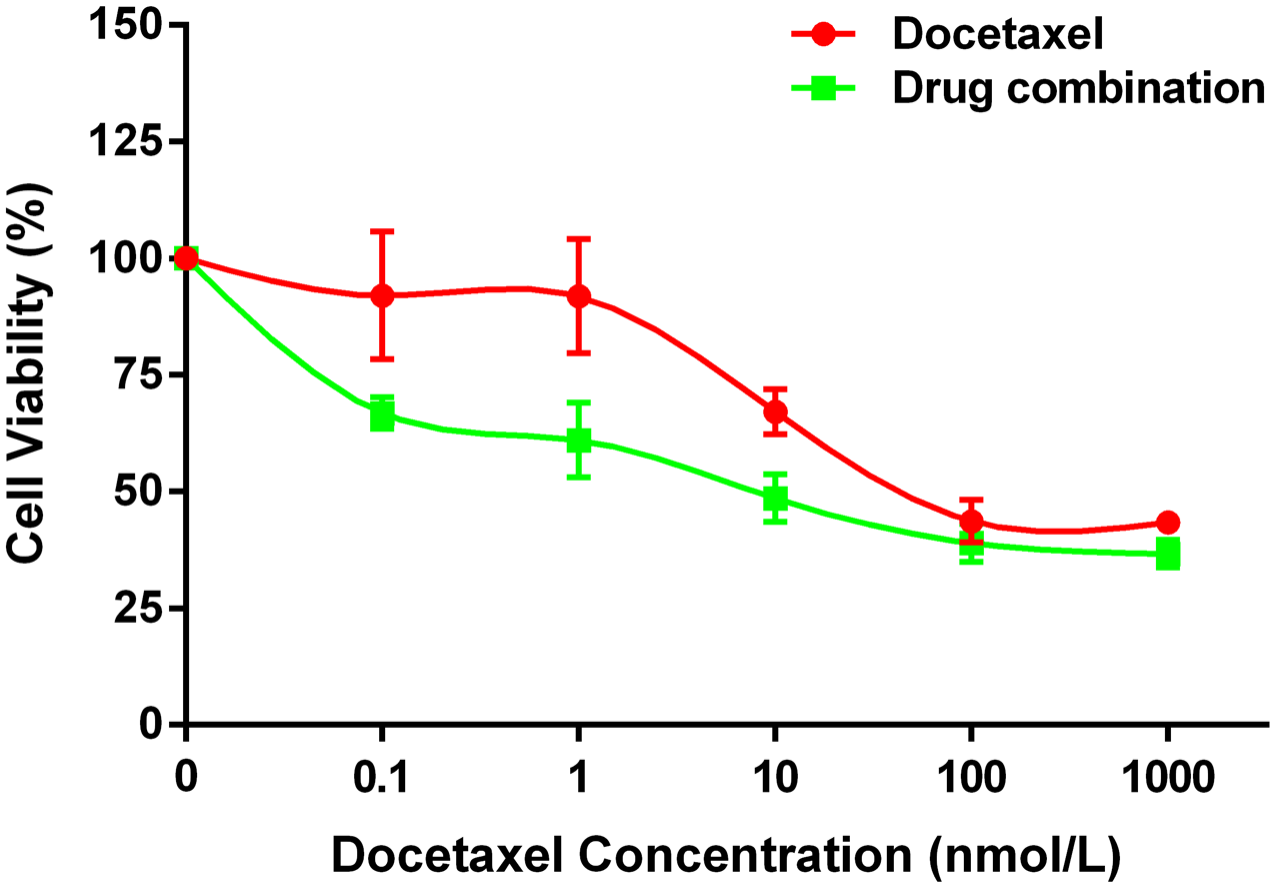
The synergistic effect of SB-3CT combined with docetaxel on PC-3 cells viability The drug combination treatment of SB-3CT and docetaxel inhibited the viability of PC-3 cells significantly compared to single drug treatment of docetaxel. Data are shown as mean±SD (* p<0.05).

Matrix metalloproteinase inhibitor (SB-3CT), docetaxel and combination treatment could reduce the viability of PC-3 cells significantly, and the viability of cells decreased gradually when the concentration of drug increased. The combination of docetaxel and SB-3CT could decrease the viability of PC-3 cells significantly compared with single drug treatment (Figure 6, P<0.001). The results of CI analysis showed when concentration of SB-3CT was 5μmol/L and 10μmol/ L, the Interactive effects of docetaxel and SB-3CT are synergism or antagonism. However, when concentration of SB-3CT was 20μmol/L, mild to moderate synergistic effects were shown (Table 1).

**Table 1:** The synergistic effect of matrix metalloproteinase inhibitor combined with docetaxel on PC-3 cells viability

SB-3CT Concentration (μmol/L)	Docetaxel (nmol/L)				
	0.1	1	10	100	1000
**5**	3.2263	2.1191	0.3592	0.7266	5.6172
**10**	31.7420	6.6903	1.0189	0.9097	6.5487
**20**	0.9652	0.8732	0.6837	0.5995	0.7743

## DISCUSSION

It was well known that early clinical stage and organ confined prostate cancer can be cured by radical surgery or radiation therapy. Hormonal therapy was one of the choices for recurrent or metastatic prostate cancer. However, hormonal therapy was not curative and the tumor always recurred after an initial androgen dependent period and inevitably progressed to castration resistant or metastatic status [14]. Unfortunately, the potential molecular mechanism of castration resistant prostate cancer or metastatic prostate cancer remained unclear. We had found CD44 could regulate glucose metabolism in small cell neuroendocrine carcinoma which may have been induced by androgen deprivation therapy [6] [15]. In this study we found PDK1 and PFKFB4, CD44 dependent metabolic genes, implicated in modulation of aggressive phenotype.

PDK1 and PFKFB4 are important enzymes regulating glucose metabolism [4]. PDK1 converts pyruvate into lactate, and elevated glycolysis may lead to acidosis which may increase invasive behavior of malignant cells [16–18]. PFKFB4 catalyzes the synthesis and degradation of fructose-2,6-bisphoshpate [19]. Silencing of PFKFB4 should divert glucose 6-phophate towards the glycolytic pathway, thereby depleting the pentose phosphate pathway [7]. We had shown CD44 knockdown in PC-3 cells results in reduced expression of PFKFB4 mRNA as well as protein levels [6]. To further investigate the regulative relationship of CD44 and key metabolic enzymes, we overexpressed CD44 in LNCaP cells by using letivirus transfection, and found overexpression of CD44 lead to increased expression levels of PDK1 and PFKFB4 which was consistent with siRNA mediated CD44 ablation assay.

As described above, PDK1 and PFKFB4 involved in the invasive behaviors in malignant cells. In this study, we found that PDK1 and PFKFB4 played a role in regulating the proliferation, invasion and migration of prostate cancer cells. It was wide accepted CD44 was a multifunctional cell membrane receptor involved in cell adhesion, tumor invasion and metastasis. In addition, strong and diffuse membrane staining for CD44 was observed in 100% of prostatic small cell neuroendocrine carcinomas, a more aggressive tumors and often present as locally advanced or metastatic disease. Most of prostatic small cell neuroendocrine carcinomas were strongly positive for PFKFB4, in contrast, the adenocarcinoma cells were negative or weakly stained [6, 20]. In this study, shRNA-mediated PDK1 and PFKFB4 ablation in prostate cancer cells should alleviates the aggressive such as cell proliferation, cell migration and invasion. Taken together, it was possible that CD44 regulates prostate cancer proliferation, invasion and migration via PDK1 and PFKFB4.

SB-3CT is a potential inhibitor of CD44. It was designed to selectively bind to the active site of gelatinases (MMP-2 and MMP-9) to block CD44 cleavage [13, 21]. It has been reported that SB-3CT is a potent inhibitor of liver metastasis and increases survival in an aggressive mouse model of T-cell lymphoma [22]. In addition, SB-3CT inhibited human prostate cancer growth and angiogenesis in a bone metastasis model [23]. In this study, we demonstrate that SB-3CT decreased glucose consumption and increased ROS levels in PC-3 cells, likely through inhibition of CD44, supporting the results of our previous study [6].

Currently the management of metastatic prostate cancer and castration resistant prostate cancer remains a complex and difficult problem because there is no curative treatment. Docetaxel-based chemotherapy became the standard treatment in patients with metastatic androgen independent prostate cancer. However, adverse events were more frequent in patients treated with docetaxel and response durations were rather short [24]. Several studies demonstrated CD44 ablation enhanced the effect of chemotherapeutic drugs in cancer cells [5, 6, 25]. In our present study, we found combination treatment of docetaxel and SB-3CT could decrease the viability of PC-3 cells significantly compared with single drug treatment. To further evaluate the combination effect, we calculated the combination index under CompuSyn software which designed follow Chou-Talalay formula [26]. The Chou-Talalay method for drug combination is based on the median-effect equation, derived from the mass action law principle, which is the unified theory that provides the common link between single entity and multiple entities, and first order and higher order dynamics. The resulting combination index theorem of Chou-Talalay offers a quantitative definition for additive effect (CI=1), antagonism (CI>1) and synergism (CI<1) in drug combinations [27]. The results of this study indicate when concentration of SB-3CT is 20μmol/L, mild to moderate synergistic effects with docetaxel are observed, which suggests combination of CD44 inhibitor and conventional chemotherapy may be the most favorable therapeutic strategy for future treatment of castration resistant or metastatic prostate cancer.

## MATERIALS AND METHODS

### Cell lines and cell culture

LNCaP and PC-3 cells were obtained from the Cell Bank of Type Culture Collection of Chinese Academy of Sciences. The cells were cultured in RPMI-1640 medium supplemented with 10% fetal bovine serum (Gibco, USA) and penicillin-streptomycin. The cells were incubated in a humidified, incubator at 37°C and 5% CO_2_.

### Plasmid preparation and transfection

shRNA sequences targeting human PDK1 or PFKFB4 were cloned into the lentiviral vector, lentiviruses were produced by transfecting 293T cells with the shRNA plasmid and packaging plasmids pGLV3/H1/GFP, PG-p1-VSVG, PG-P2-REV and PG-P3-RRE. Supernatants containing lentiviral particles were collected 72 hours after transfection, filtered through a 0.45 μm filter and stored at -80°C. LNCaP and PC-3 cells were infected with lentiviral particles containing the indicated shRNA for 24 hours. Fresh medium was changed and the cells were cultured for 72 hours before being used in experiments. With similar methods, the lentivirus expressing CD44 homo gene was transduced into LNCaP cells to generate stable expression of CD44 LNCaP cells.

### RNA interference

CD44 was knocked down with small interfering RNA (siRNA) using DharmaFECT transfection reagents (Thermo Scientific, USA). PC-3 cells were transfected with 20 nM of CD44 siRNA or control siRNA (Intergrated DNA Technologies, USA) for 48 hours.

### Western blot

As previous described [28], briefly, cell extracts were prepared with lysis buffer supplemented with protease inhibitor (Beyotime, China). Equal amounts of proteins were separated on 8% SDS-polyacrylamide gels followed by transfer to polyvinylidene fluoride membrane (Bio-Rad, USA). After blocking with non-fat milk, the membrane is incubated with diluted primary antibody (1:500; Abcam, UK), followed by secondary antibody (Sigma, USA) incubation. The proteins were visualized by ECL Plus kit (Pierce, USA).

### Quantitative real time polymerase chain reaction analysis

QRT-PCR was carried out using standard techniques. Total RNA was extracted by following the RNAiso Plus instruction (TaKaRa, JPN), Reverse transcription was performed using the PrimeScript RT-PCR system (Takara, JPN). The internal control was β-actin. Data were normalized by the amount ofβ-actin mRNA. Expression of relative genes was calculated using 2−ΔΔCt methods. The following specific forward and reverse primers were used:

**Table:** 

CD44:	forward	ACCACGGGCTTTTGACCA,
	reverse	GGTGAATGAGGG
		GAGGGTG;
PDK1:	forward	GAAGATGAGTGAC
		CGAGGAGG,
	reverse	GTAAAGACGTGAT
		ATGGGCAATC
PFKFB4:	forward	TTAATTTTGGAGAAC
		AGAATGGC
	reverse	CGTAGCCTCATCACT
		GTCGC
β-actin:	forward	TGACGTGGACATCC
		GCAAAG
	reverse	CTGGAAGGTGGACA
		GCGAGG

### Cell proliferation assay

Cell proliferation was quantified using MTT assay kit (Sigma, USA) according to the manufacturer’s protocols. The absorbance was determined at time point of 24, 48 and 72 hours after seeding cells. This experiment was done in triplicate wells.

### Cell invasion assay

Cell Invasion was determined by transwell assay. In brief, the upper chamber surface of transwell membrane (Costar, USA) with a 12-μm pore size was coated with matrigel (BD Biosciences, USA) and dried at room temperature overnight. Cell suspension were added to Matrigel-coated transwell in the bottom well filled with culture medium. The lower side of the transwell membranes were fixed and stained with trypan blue. The numbers of migrated cell were counted in 5 random high power fields (HPF), and determined the average cell number migrated per HPF.

### Cell migration assay

Cell migration was measured with wound healing assay. The cells were seeded in six well plate and incubated in a humidified, incubator at 37°C and 5% CO_2_. When the cells reached 80% confluence, a wound field was made using a sterile 200μL pipette tip. The scratched cells were removed by washing the plate for three times. The mobilized cells were observed by a digital camera system after 24, 48 and 72hours. Migration distance was calculated applying the software program HMIAS-2000.

### Measurement of glucose consumption and lactate secretion

The methods had been described in our previous study [6]. In brief, PC-3 cells were seeded and incubated in low glucose RPMI-1640 medium. Glucose concentrations of the media were measured with glucose assay kit (Sigma, USA).

Lactate concentration of media was determined by using an enzymatic method [29]. Briefly, the hydrazine buffer, NAD and LDH (Sigma) were added in media. Absorbance at 340nm was measured to determine the lactate levels. Distilled water was used as blanks.

### Measurement of reactive oxygen species (ROS)

As previous described [6], intracellular ROS levels were determined with H2DCF-DA (Invitrogen, USA). Cells were seeded in 96-well black assay plate (Corning, USA), and incubated with serum-free media containing 10μmol/L H2DCF-DA for 30min. ROS levels were measured at excitation and emission wavelengths of 485 and 520 nm using a Synergy H1 Hybrid multi-mode microplate reader (Bio-Tek Instruments, USA). Control media without dye were used as background fluorescence. Cell numbers were normalized before measurement.

### Statistical analysis

All experiments were performed in triplicates. The data were presented as mean±SD. Statistical significance was determined using paired or independent sample t-test or ANOVA. *P* value less than 0.05 was considered to be statistically significant. Statistical calculations were carried out by Microsoft excel 2007 or SPSS 19.0. Combination index (CI), a measure of synergistic activity was calculated with CompuSyn 1.0 software.

## References

[1] Siegel RL, Miller KD, Jemal A (2016). Cancer statistics, 2016. CA Cancer J Clin.

[2] Chen W, Zheng R, Baade PD, Zhang S, Zeng H, Bray F, Jemal A, Yu XQ, He J (2016). Cancer statistics in China, 2015. CA Cancer J Clin.

[3] Warburg O, Wind F, Negelein E (1927). The metabolism of tumors in the body. J Gen Physiol.

[4] Miletti-González KE, Murphy K, Kumaran MN, Ravindranath AK, Wernyj RP, Kaur S, Miles GD, Lim E, Chan R, Chekmareva M, Heller DS, Foran D, Chen W (2012). Identification of function for CD44 intracytoplasmic domain (CD44-ICD): modulation of matrix metalloproteinase 9 (MMP-9) transcription via novel promoter response element. J Biol Chem.

[5] Tamada M, Nagano O, Tateyama S, Ohmura M, Yae T, Ishimoto T, Sugihara E, Onishi N, Yamamoto T, Yanagawa H, Suematsu M, Saya H (2012). Modulation of glucose metabolism by CD44 contributes to antioxidant status and drug resistance in cancer cells. Cancer Res.

[6] Li W, Cohen A, Sun Y, Squires J, Braas D, Graeber TG, Du L, Li G, Li Z, Xu X, Chen X, Huang J (2016). The Role of CD44 in Glucose Metabolism in Prostatic Small Cell Neuroendocrine Carcinoma. Mol Cancer Res.

[7] Ros S, Santos CR, Moco S, Baenke F, Kelly G, Howell M, Zamboni N, Schulze A (2012). Functional metabolic screen identifies 6-phosphofructo-2-kinase/fructose-2,6-biphosphatase 4 as an important regulator of prostate cancer cell survival. Cancer Discov.

[8] Tai S, Sun Y, Squires JM, Zhang H, Oh WK, Liang CZ, Huang J (2011). PC3 is a cell line characteristic of prostatic small cell carcinoma. Prostate.

[9] Fujiwara S, Kawano Y, Yuki H, Okuno Y, Nosaka K, Mitsuya H, Hata H (2013). PDK1 inhibition is a novel therapeutic target in multiple myeloma. Br J Cancer.

[10] Xian ZY, Liu JM, Chen QK, Chen HZ, Ye CJ, Xue J, Yang HQ, Li JL, Liu XF, Kuang SJ (2015). Inhibition of LDHA suppresses tumor progression in prostate cancer. Tumour Biol.

[11] Gatenby RA, Gillies RJ (2004). Why do cancers have high aerobic glycolysis?. Nat Rev Cancer.

[12] Ranasinghe HS, Scheepens A, Sirimanne E, Mitchell MD, Williams CE, Fraser M (2012). Inhibition of MMP-9 activity following hypoxic ischemia in the developing brain using a highly specific inhibitor. Dev Neurosci.

[13] Thorne RF, Legg JW, Isacke CM (2004). The role of the CD44 transmembrane and cytoplasmic domains in co-ordinating adhesive and signalling events. J Cell Sci.

[14] Li Z, Chen CJ, Wang JK, Hsia E, Li W, Squires J, Sun Y, Huang J (2013). Neuroendocrine differentiation of prostate cancer. Asian J Androl.

[15] Lipianskaya J, Cohen A, Chen CJ, Hsia E, Squires J, Li Z, Zhang Y, Li W, Chen X, Xu H, Huang J (2014). Androgen-deprivation therapy-induced aggressive prostate cancer with neuroendocrine differentiation. Asian J Androl.

[16] Smallbone K, Gavaghan DJ, Gatenby RA, Maini PK (2005). The role of acidity in solid tumour growth and invasion. J Theor Biol.

[17] Montcourrier P, Mangeat PH, Valembois C, Salazar G, Sahuquet A, Duperray C, Rochefort H (1994). Characterization of very acidic phagosomes in breast cancer cells and their association with invasion. J Cell Sci.

[18] Gillies RJ, Robey I, Gatenby RA (2008 (Suppl 2)). Causes and consequences of increased glucose metabolism of cancers. J Nucl Med.

[19] Minchenko OH, Ochiai A, Opentanova IL, Ogura T, Minchenko DO, Caro J, Komisarenko SV, Esumi H (2005). Overexpression of 6-phosphofructo-2-kinase/fructose-2,6-bisphosphatase-4 in the human breast and colon malignant tumors. Biochimie.

[20] Simon RA, di Sant’Agnese PA, Huang LS, Xu H, Yao JL, Yang Q, Liang S, Liu J, Yu R, Cheng L, Oh WK, Palapattu GS, Wei J, Huang J (2009). CD44 expression is a feature of prostatic small cell carcinoma and distinguishes it from its mimickers. Hum Pathol.

[21] Gialeli C, Theocharis AD, Karamanos NK (2011). Roles of matrix metalloproteinases in cancer progression and their pharmacological targeting. FEBS J.

[22] Krüger A, Arlt MJ, Gerg M, Kopitz C, Bernardo MM, Chang M, Mobashery S, Fridman R (2005). Antimetastatic activity of a novel mechanism-based gelatinase inhibitor. Cancer Res.

[23] Bonfil RD, Sabbota A, Nabha S, Bernardo MM, Dong Z, Meng H, Yamamoto H, Chinni SR, Lim IT, Chang M, Filetti LC, Mobashery S, Cher ML, Fridman R (2006). Inhibition of human prostate cancer growth, osteolysis and angiogenesis in a bone metastasis model by a novel mechanism-based selective gelatinase inhibitor. Int J Cancer.

[24] Tannock IF, de Wit R, Berry WR, Horti J, Pluzanska A, Chi KN, Oudard S, Théodore C, James ND, Turesson I, Rosenthal MA, Eisenberger MA (2004). TAX 327 Investigators. Docetaxel plus prednisone or mitoxantrone plus prednisone for advanced prostate cancer. N Engl J Med.

[25] Thapa R, Wilson GD (2016). The Importance of CD44 as a Stem Cell Biomarker and Therapeutic Target in Cancer. Stem Cells Int.

[26] Chou TC, Talalay P (1984). Quantitative analysis of dose-effect relationships: the combined effects of multiple drugs or enzyme inhibitors. Adv Enzyme Regul.

[27] Chou TC (2010). Drug combination studies and their synergy quantification using the Chou-Talalay method. Cancer Res.

[28] Li Z, Sun Y, Chen X, Squires J, Nowroozizadeh B, Liang C, Huang J (2015). p53 Mutation Directs AURKA Overexpression via miR-25 and FBXW7 in Prostatic Small Cell Neuroendocrine Carcinoma. Mol Cancer Res.

[29] Lima JL, Lopes TI, Rangel AO (1998). Enzymatic determination of L () lactic and L(-) malic acids in wines by flow-injection spectrophotometry. Anal Chim Acta.

